# Effectively predicting HIV-1 protease cleavage sites by using an ensemble learning approach

**DOI:** 10.1186/s12859-022-04999-y

**Published:** 2022-10-27

**Authors:** Lun Hu, Zhenfeng Li, Zehai Tang, Cheng Zhao, Xi Zhou, Pengwei Hu

**Affiliations:** 1 Xinjiang Technical Institute of Physics and Chemistry, Chinese Academy of Sciences, Ürümqi, China; 2 School of Computer Science and Artificial Intelligence, Wuhan University of Technology, Wuhan, China

**Keywords:** HIV-1 protease, Cleavage sites prediction, Asymmetric bagging, Biased SVM, Ensemble learning

## Abstract

**Background:**

The site information of substrates that can be cleaved by human immunodeficiency virus 1 proteases (HIV-1 PRs) is of great significance for designing effective inhibitors against HIV-1 viruses. A variety of machine learning-based algorithms have been developed to predict HIV-1 PR cleavage sites by extracting relevant features from substrate sequences. However, only relying on the sequence information is not sufficient to ensure a promising performance due to the uncertainty in the way of separating the datasets used for training and testing. Moreover, the existence of noisy data, i.e., false positive and false negative cleavage sites, could negatively influence the accuracy performance.

**Results:**

In this work, an ensemble learning algorithm for predicting HIV-1 PR cleavage sites, namely EM-HIV, is proposed by training a set of weak learners, i.e., biased support vector machine classifiers, with the asymmetric bagging strategy. By doing so, the impact of data imbalance and noisy data can thus be alleviated. Besides, in order to make full use of substrate sequences, the features used by EM-HIV are collected from three different coding schemes, including amino acid identities, chemical properties and variable-length coevolutionary patterns, for the purpose of constructing more relevant feature vectors of octamers. Experiment results on three independent benchmark datasets demonstrate that EM-HIV outperforms state-of-the-art prediction algorithm in terms of several evaluation metrics. Hence, EM-HIV can be regarded as a useful tool to accurately predict HIV-1 PR cleavage sites.

## Introduction

Acquired immunodeficiency syndrome (AIDS) is caused by human immunodeficiency virus type 1 (HIV-1) [1], which destroys the immune system by attacking T-cells in the body. Therefore, inhibiting the replication of HIV-1 is of great significance for designing effective anti-AIDS drugs [2]. A series of biological experiments have been carried out in order to better understand the replication mechanism of HIV-1 [3–5], and their results show that HIV-1 protease cleaves the polyproteins at multiple sites to generate mature and infectious virus particles. Therefore, an effective way to treat AIDS is to inhibit the activity of the corresponding HIV-1 protease by preventing the replication of HIV-1 [6].

HIV-1 protease inhibitors (HIV-1 PI) hinder the normal function of HIV-1 protease by tightly binding to the substrates of HIV-1 protease [7]. It is for this reason that predicting the cleavage site of HIV protease substrate is important for the design of effective HIV-1 PIs. In addition, understanding the substrate specificity of HIV-1 protease can effectively reduce the side effects caused by HIV-1 PIs [8]. However, only resting on existing biological knowledge is difficult to accurately and efficiently verify the existence of cleavage sites in the HIV-1 protease substrates [9], and as a result it is still a challenging problem to determine the substrate specificity of HIV-1 protease. Although researchers have conducted laboratory-based experiments to determine the cleavage site of HIV-1 protease substrates, they suffer the disadvantages of being time-consuming and labor-intensive [10].

With the development of machine learning techniques in bioinformatics [11], a variety of machine learning-based methods have been developed to effectively predict the existence of HIV-1 protease cleavage sites in the substrates [12–31]. They usually regard the prediction problem as a typical binary classification task, which is then achieved with a two-step procedure. First, relevant features are extracted from substrate sequences in different ways, and they are used to construct the feature vectors of octamers. After that, these feature vectors are taken as input for selected classification models so as to complete the prediction task. Although there is little relevant biological knowledge indicating the association between extracted features and HIV-1 protease specificity, these computational methods have demonstrated their wide availability and generally satisfactory predictive performance for large-scale prediction of HIV-1 protease cleavage sites [21].

In the context of supervised learning, the quality of datasets plays a critical role in determining the performance of predication algorithms [32]. Although cleavable octamers have already been verified through expensive and time-consuming biological experiments, uncleavable octamers are artificially generated by using different strategies for performance evaluation. Obviously, there are two problems regarding benchmark datasets obtained in this way. First, since cleavable octamers are only a small part of all octamers, the number of uncleavable octamers in benchmark datasets is usually much larger than that of cleavable octamers. Second, the artificially generated uncleavable octamers have not been verified by laboratory experiments, and there may exist some cleavable octapeptides that are falsely grouped in this class. Therefore, when we directly apply specific classifiers to predict HIV-1 protease cleavage sites, several issues regarding the imbalance and noisy data certainly affect the prediction performance. Taking the imbalance between cleavable and uncleavable octamers as an example, the number of cleavable octamers is normally much smaller than that of uncleavable octamers, and accordingly the trained prediction model is more biased towards to the majority class, thus leading to the poor performance in predicting cleavable octamers [33]. In addition, the existence of false-negative data in the uncleavable octamers also degrades the prediction accuracy of classifiers.

According to our practical study on the existing benchmark datasets collected for HIV-1 protease cleavage site prediction, we realize that effectively dealing with the imbalance and false-negative instances in the training dataset is essential to obtain an efficient and accurate prediction model. As suggested by many studies [34–36], the imbalance factor affects the prediction performance biased towards the majority class. Hence, most of existing computational models on the HIV-1 problem demonstrate their promising ability in identifying uncleavable octamers, and accordingly they achieve better performance in terms of AUC. However, regarding the prediction of HIV-1 cleavage sites, what we are most interested in is to accurately identifying cleavable sites from HIV-1 protease substrates. In this regard, we introduce the imbalance issue to alleviate the bias towards to the majority class.

To this end, we propose an ensemble learning model, namely EM-HIV, which target to integrate asymmetric bagging [37] with biased SVM classifiers to reduce the impact of imbalance and false-negative instances on the prediction model. By doing so, a more accurate prediction model can thus be constructed for predicting HIV-1 protease cleavage sites, which consist of the minority class in benchmark datasets. The consideration of adopting asymmetric bagging is to keep the positive instances in the training dataset unchanged, and we only resample from the negative instances to ensure the balance in the subsets. At the same time, biased SVM classifier assigns different error weights to positive and negative instances such that EM- HIV is more interested in predicting cleavable octamers. In addition, EM- HIV combines amino acid identity, chemical group properties, and variable-length coevolutionary patterns to construct feature vectors of octamers. This allows EM-HIV to make full use of the sequence information of substrates for the prediction task. To verify the performance of EM-HIV in predicting HIV-1 protease cleavage site, a series of extensive experiments have been conducted by comparing it with several state-of-the-art prediction models.

## Related work

In the early stage of studies on predicting HIV-1 protease cleavage sites, much attention has been attracted on using different classification models by considering the prediction task as a non-linear problem [16]. In particular, Thompson et al. [12] apply an artificial neural network (ANN) with a standard feed-forward multilayer perceptron (MLP) to predict HIV-1 protease cleavage sites, and evaluate the performance on a small set of octapeptides. Later, Cai et al. [13] repeat Thompson’s work on a new dataset using a standard MLP with eight hidden units. The results indicate that MLP has superior performance in dealing with non-linear problems, such as the prediction of HIV-1 protease cleavage sites. Cai et al. [14] further apply SVM with different kernel functions to predict HIV-1 protease cleavage sites, and find that the SVM classifier with a Gaussian kernel function performs better in the experiments. This fact also implies the strong predictive ability of SVM on nonlinear problems. Narayanan et al. [15] attempt to use a decision tree for predicting HIV-1 protease cleavage sites, but they conclude that the performance is always inferior to the ANN. Kontijevkis et al. [17] collect benchmark datasets from HIV proteomic studies, and then design a rule-based prediction model based on the rough set theory to analyze the specificity of HIV-1 protease substrates. Their experimental results indicate that the cleavability of substrates would be stronger if at least three amino acids are combined in the substrate sequences. HIVcleave [18] establishes the first web server to provide the online service of predicting the HIV-1 protease cleavage sites, and it combines the discriminant function algorithm and the vectorized sequence-coupling model to complete the prediction task.

With the increase of verified cleavable octamers, it has been pointed out by [16] that the HIV-1 protease cleavage site prediction should be a linear problem, and the consideration of linear classifiers could lead better performance on this task. Following this motivation, more attention has been attracted on how to extract linearly separable features from substrate sequences. Li et al. [19] develop a theoretical framework based on the kernel method, which projects octamers onto the local kernel space to reduce the dimensionality of resulting features. A linear SVM classifier is then adopted to predict HIV-1 protease cleavage sites. Gok et al. [20] study several different coding schemes, and propose an OETMAP coding scheme based on amino acid features to complete the prediction task. Then validation experiments are conducted with standard amino acid encodings on two benchmark datasets, and the results verify that the OETMAP coding method effectively improves the prediction performance. Rognvaldsson et al. [21] propose a prediction method combining orthogonal coding and linear SVM, and they claim that this combination may be the best predictor. Utilizing the area under receiver operating characteristics (AUC) as a fitness measure for the evaluation of optimal ensemble, an optimal ensemble formation technique is proposed to solve the prediction problem of HIV-1 cleavage sites by using seven encoding techniques and four SVM kernels [22]. PROSPERous [23], as a feature-based integrated system, uses substrate sequences and structural features to design different scoring functions for feature vector construction, and then adopts the logistic regression model to predict the HIV-1 protease cleavage site. Singh et al. [24] adopt a cross-domain approach by incorporating the characteristics extracted from various amino acid encoding techniques such that the impact of insufficient training data could be alleviated. For improved prediction performance, a cognitive framework using evolutionary intelligence is proposed by adaptively determining the ideal parameter values for selected kernels [25].

As a new integrated prediction model, iProt-Sub [26] first combines heterogeneous features and structural features, and then adopts a two-step feature selection procedure to improve the model’s accuracy by eliminating redundant and irrelevant features. Following the coevolution observed in residuals, EvoCleave [27] targets to extract features based on the coevolutionary information of substrate sequences. The experimental results show that EvoCleave is very promising in predicting novel HIV-1 protease cleavage sites. Based on the coevolutionary patterns proposed by EvoCleave, Li and Hu [28] further propose EvoCleave V2.0 to identify variable-length coevolutionary patterns from substrate sequences. The results of 10-fold cross-validation experiments demonstrate that EvoCleave V2.0 is more accurate for the prediction task. Since optimization techniques have been widely adopted to effectively solve many practical applications [38], a multiobjective evolutionary-based multi-kernel model [29] is proposed by formulating the HIV-1 protease cleavage site prediction problem into a bi-objective optimization problem. Combining the knowledge from experimental studies, a multitask learning model is developed recently based on multi-kernel [30], and it utilizes the dependencies among various related tasks to build a stronger predictive model for HIV-1 protease cleavage sites prediction. Since certain noisy can be contained by mislabeling cleavable octamers as negative instances, PU-HIV [31] considers unknown substrate sites as unlabeled samples, and makes use of positive-unlabeled learning to effectively predict HIV-1 protease cleavage sites.

**Fig. 1:**
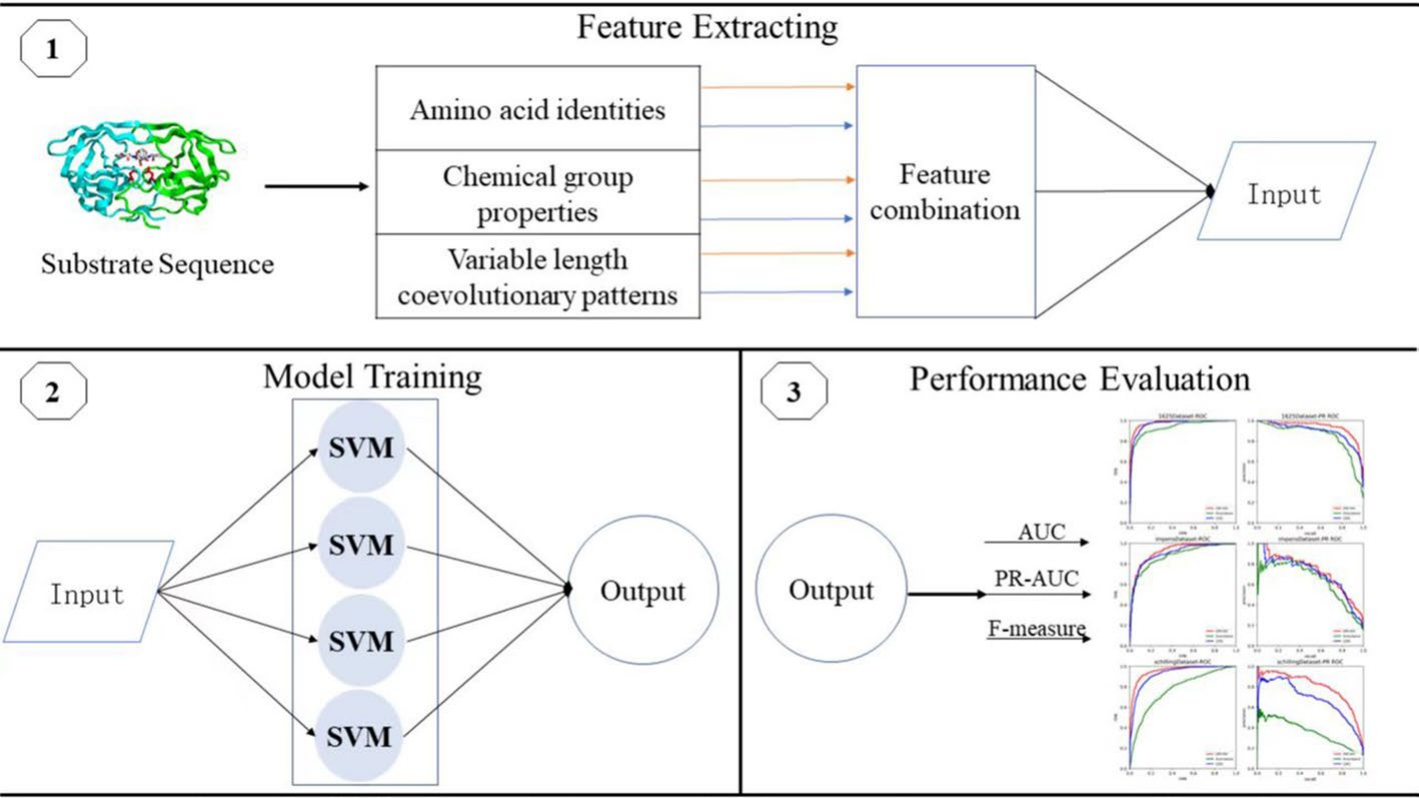
The pipeline of our study

## Materials and methods

The structure of this section consists of the following three steps. The first step is to extract features of amino acids from the perspectives of amino acid identities, chemical group properties and variable-length coevolutionary patterns, and these feature are then used to construct the feature vector for each octamer accordingly. In the second step, the proposed model, i.e., EM-HIV, is trained by combining the idea of asymmetric bagging with biased SVM. Last, we adopt different evaluation metrics to assess the performance of EM-HIV. Figure 1 shows the pipeline of these three steps.

### Feature extraction

Each octamer is a sequence composed of eight amino acids. In particular, given an alphabet set $\Lambda =\lbrace \lambda _i\rbrace (1\le i\le n_\Lambda , n_\Lambda =20)$ representing a set of 20 distinct amino acids, $\Gamma =\{\beta _j \vert \beta _j = \lambda _m \lambda _n\}(1 \le j \le n_\Gamma ^2, 1\le m,n \le n_\Gamma )$ is composed of a total of 400 different amino acid sequences with length 2, and an octamer is represented as ${\textbf {P}}=P_1 P_2 P_3 P_4 P_5 P_6 P_7 P_8$ where $P_i \in \Lambda (1 \le i \le 8)$. In order to use machine learning methods for predicting the cleavage site of HIV-1 protease, each octamer needs to be mapped to an *N*-dimensional feature vector. In this work, three different kinds of characteristics, i.e., amino acid identities, chemical group properties, and variable length coevolutionary patterns are used to extract features from octamers. This step makes full use of substrate sequence information for the prediction task.

#### Amino acid identities

Amino acid identities are based on the amino acids of octamers. Each amino acid is mapped to a 20-dimensional vector with an orthogonal coding scheme. In this regard, each octamer is mapped to an $8 \times 20$ matrix, which is transformed into a 160-dimensional vector in the feature space. However, since amino acids at different positions are independent, the value of the last position can thus be limited by the other elements so that the feature dimension can be simplified to 152 dimensions without considering the last position.

**Table 1:** The chemical classes of amino acids

Chemical group	Amino acids
Sulfur-containing	C, M
Aliphatic 1	A, G, P
Aliphatic 2	I, L, V
Acidic	D, E
Basic	H, K, R
Aromatic	F, W, Y
Amide	N, Q
Small hydroxy	S, T

#### Chemical group properties

In addition to amino acid identities, the chemical group properties of amino acids are also considered for constructing the feature vectors of octamers. To do so, $\Lambda$ is first divided into eight independent chemical groups [39]. The detailed division information is presented in Table 1. The construction process is similar to that of amino acid identities. In particular, each amino acid is mapped into an 8-dimensional feature vector using an orthogonal coding scheme due to the independence of chemical groups. One should note that an amino acid can only belong to one chemical group. Therefore, the last amino acid in an octamer is restricted by the amino acids in the other positions, and the length of feature vectors can thus be reduced to 7. The total number of features extracted from the chemical properties is 8 $\times$ 7=56 for all octamers.

#### Variable length coevolutionary patterns

According to our previous studies [40, 41], the fact that amino acids located at different residues might co-evolve is of great significance for sequence analysis. Inspired by this observation, EvoCleave V2.0 is proposed in [28] to extract variable-length coevolutionary patterns for better characterizing octamers. In this work, we inotruce three different kinds of coevolutionary patterns including A_A, A_AB and AB_A. Take A_AB as an example, $(\lambda _i, \beta _j)_k$ denotes that $\lambda _i$ is followed by $\beta _j$ at $k-1$ positions later, and EvoCleave V2.0 then determines whether $(\lambda _i, \beta _j)_k$ is a coevolutionary pattern by (1).

1 $$\begin{aligned} diff\big ((\lambda _i,\beta _j)_k\big )=\frac{p\big ((\lambda _i,\beta _j)_k\big ) -p\big ((\lambda _i,*)_k\big )p\big ((*,\beta _j)_k\big )}{\sqrt{\frac{p\big ((\lambda _i,*)_k\big ) p\big ((*,\beta _j)_k\big )}{n_1}\Big (1-p\big ((\lambda _i,*)_k\big )\Big )\Big (1-p\big ((*,\beta _j)_k\big )\Big )}} \end{aligned}$$

In the above equation, $p\big ((\lambda _i,\beta _j)_k\big )$, $p\big ((\lambda _i,*)_k\big )$ and $p\big ((*,\beta _j)_k\big )$ are the respective probabilities that $(\lambda _i, \beta _j)_k$, $(\lambda _i, *)_k$ and $(*, \beta _j)_k$ are observed in octamers, and $n_1$ is the number of octamers. It should be noted that the octamers mentioned here only refer to those that are cleavable. Since the value of *diff* follows a normal distribution, $(\lambda _i, \beta _j)_k$ is considered as a coevolutionary pattern in $n_1$ at a confidence level of 95% if $diff\big ((\lambda _i,\lambda _j)_k\big ) \ge 1.96$. EvoCleave V2.0 then uses (2) to quantify the amount of evidence provided by each coevolutionary pattern from the perspective of mutual information.

2 $$\begin{aligned} weight\big ((\lambda _i,\beta _j)_k\big )= \log \frac{p\big ((\lambda _i,\beta _j)_k\big )}{p\big ((\lambda _i,*)_k\big )p\big ((*,\beta _j)_k\big )} -\log \frac{p\big ((\lambda _i,*)_k\big )-p\big ((\lambda _i,\beta _j)_k\big )}{p\big ((\lambda _i,*)_k\big )(1-p\big ((*,\beta _j)_k\big )} \end{aligned}$$

### Model training

#### Support vector machines

Support vector machine (SVM) [42] is a popular classification model and has been widely used in many applications across different research fields. It is a linear classifier with the largest interval defined in the feature space, and can effectively handle high dimensional datasets and nonlinear classification using kernel functions. A classic SVM classifier constructs a hyperplane in the feature space to distinguish between positive and negative instances for binary classification.

For a given training set $D=\big \lbrace (p_{i}, y_{i})\big \rbrace (1\le i\le n)$ where $p_i$ denotes the N-dimensional feature vector of $P_i$ and $y_i \in \lbrace -1,1\rbrace$ is its label, SVM intends to find a hyperplane($\omega ^T p_i+b=0$) that correctly distinguishes positive and negative instances. Assuming that the first $m-1$ octamers in *D* are positive instances labeled as $y_{i}=1(1 \le i\le m-1)$, while the rest are negative with labels set to -1. However, instead of using classic SVM as the weak learner, we decide to used its biased variant [43] for model training to alleviate the impacts of imbalance and false-negative instances in the benchmark dataset. A biased SVM with two L1-norm soft margins is defined as:

3 $$\begin{aligned} & Minimize:\quad ~\frac{1}{2}\omega ^{T} \omega + C_{1} \sum\limits_{{i = 1}}^{{m - 1}} {\xi _{i} } + C_{2} \sum\limits_{{i = m}}^{n} {\xi _{i} } \\ & s.t.~\;y_{i} (\omega ^{T} p_{i} + b) \ge 1 - \xi _{i} ,\xi _{i} \ge 0,i = 1,2, \ldots ,n \\ \end{aligned}$$

where $\omega$ is the normal vector of hyperplane, $\xi$ refers to the corresponding slack variable used to calculate the error cost, *b* represents the offset of hyperplane from the origin along $\omega$, $C_1$ and $C_2$ are the penalty parameters of the training errors in misidentifying positive and negative samples respectively. Based on the soft margin, we incorporate the linear kernel function defined by (4) into (3) to predict HIV-1 protease cleavage sites. In (3), the performance of a biased SVM can be fine-tuned by adjusting the values of $C_1$ and $C_2$.

4 $$\begin{aligned} kernel(p_i,p_j)=p_i^T\cdot p_j \end{aligned}$$

When training a biased SVM classifier, we strictly follow the instruction provided in [21] to determine the optimal value of $C_1$, which is varied over the set $\lbrace 2^{{-}5}, 2^{{-}4},2^{{-}3},\cdots , 2^{5}\rbrace$. For the value of $C_2$, we set it by (5), and the values of $\beta$ are varied from the set $\lbrace 2, 5, 10, 20, 30, 50, 100, 200\rbrace$. Obviously, given a predetermined $C_1$, the value of $C_2$ decreases when a larger integer is assigned to $\beta$. After evaluating all possible combinations of $C_1$ and $C_2$, we use the combination with the best performance as the final setting to train the biased SVM classifier for predicting HIV-1 PR cleavage sites.

5 $$\begin{aligned} C_2=\frac{C_1}{\beta } \end{aligned}$$

#### Asymmetric bagging

Asymmetric bagging Bagging [37] is a popular ensemble model that combines bootstrapping and aggregation advantages. The original Bagging algorithm [44] is mainly divided into two steps. First, a bootstrapping process is applied by randomly constructing multiple training subsets from the original training set. Second, a weak learner is trained for each subset, and then an aggregation of results from all learners is performed with simple strategies. For a weak learner, although the bagging algorithm can improve its prediction robustness, the imbalanced issue may also degrade its generalization ability. Obviously, it is unreasonable to directly use the bagging algorithm for predicting the HIV-1 protease cleavage sites, as the number of cleavable octamers in the benchmark dataset is much smaller than that of uncleavable octamers. For this reason, we adopt an asymmetric bagging strategy to solve the imbalance problem. In particular, asymmetric bagging only performs bootstrapping on the negative instances while preserving all positive instances. For each subset, the number of selected negative instances is equivalent to that of positive ones in the benchmark dataset. This ensures that each weak learner is trained in balance environment, thus reducing the impact of imbalance issue.
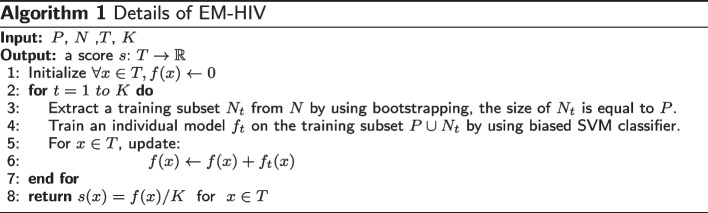


One should note that in the procedure of asymmetric bagging, it is also possible for EM-HIV to produce bias towards learning the positive instances, but such a bias is more trivial than that towards learning the negative instances, which belong to the majority class in our datasets. Regarding the setting of weak learners, we intend to assign a larger value to $C_{1}$ and a smaller one to $C_{2}$ in order to decrease the sensitivity of SVM against negative samples. This setting ensures that our training model can classify positive examples more correctly, reducing the impact of false negative data on prediction accuracy.

In summary, the details of how to train EM-HIV by using asymmetric bagging and biased SVM is described in Algorithm 1, where *P* is the set of positive instances in the training dataset, *N* is the set of negative instances in the training dataset, *T* is a independent query instance for testing, and *K* is the number of weak learners. Intuitively, a larger value of *K* is more likely to yield a better prediction performance. Finally, we average the prediction results obtained from all *K* weak learners to make the final prediction.

### Performance evaluation

After obtaining the prediction scores of all octamers in the testing dataset, several independent metrics, i.e., AUC, the area under the Precision–Recall curve (PR-AUC) and F-measure, are used to evaluate the prediction performance.

## Experiments

To evaluate the performance of EM-HIV in predicting HIV-1 protease cleavage sites, we have conducted a series of extensive experiments and compared it with several state-of-the-art prediction models, including HIVcleave [18], Rognvaldsson et al. [21], PEOSPERous [23], iProt-Sub [26] and EvoCleave [27]. All these models except iProt-Sub extract relevant features from substrate sequences, while iProt-Sub integrates different biological information to train classifiers.

### Benchmark datasets

To evaluate the performance and performance of EM-HIV, we select three frequently used and independent datasets in the experiments to avoid the bias caused by the selection of training data. Detailed descriptions about these datasets are shown in Table 2. Among them, both 1625Dataset and impensDataset are linearly separable while schillingDataset is non-linearly separable. Downloadable resources for these datasets are available in our GitHub repository. It is noted that a common characteristic of these datasets is the imbalance between cleavable and uncleavable octamers, as the number of uncleavable octamers is much more than that of cleavable octamers.

**Table 2:** Detailed descriptions of benchmark datasets

Dataset	Source	Octamers	Cleaved	Uncleaved
1625Dataset	[17]	1625	374	1251
impensDataset	[21]	947	149	798
schillingDataset	[21]	3272	434	2838

The column of Source gives the original source of corresponding dataset. The column of Octamers is the number of all octamers. The columns of Cleaved and Uncleaved are the respective numbers of positive and negative instances

### Evaluation metrics

There are three different evaluation metrics adopted to quantitatively indicate the superiority of EM-HIV, and they are the area under the receiver operating characteristics curve (AUC), the area under the Precision–Recall receiver operating characteristics curve (PR-AUC) and F-measure. Among them, AUC considers the prediction accuracy as a trade-off between sensitivity and specificity given different thresholds, but its scores may lead to an over-optimistic conclusion on imbalanced datasets [45]. Hence, in addition to AUC, we also adopt PR-AUC that is more proper to alleviate the bias towards the majority class. As a popular metric for binary classification problems, F-measure indicates the harmonic mean of Precision and Recall. The details of computing F-measure can be found in [31].

### 10-fold cross validation

To avoid bias resulted from random selection and obtain more reliable experimental results, 10-fold cross-validation (CV) scheme is used for performance evaluation. To do so, we first divide the benchmark dataset into 10 folds with equal size. For each CV, a fold is selected as the test data while the rest are used for training EM-HIV. This process is repeated for 10 times by alternatively taking each fold as the test data.

**Table 3:** Experiment results of 10-fold CV

Dataset	Model	AUC	PR-AUC	F-measure		
				Precision	Recall	F-measure
1625Dataset	EM-HIV	**0.98**	**0.94**	0.82	0.91	**0.86**
	EvoCleave	0.93	0.84	0.85	0.74	0.8
	Rognvaldsson et al.	0.97	0.9	0.85	0.8	0.83
	PEOSPERous	0.82	0.33	0.23	1	0.38
	HIVcleave	0.73	0.61	0.69	0.67	0.68
	iProt-Sub	0.68	0.41	0.41	0.26	0.32
impensDataset	EM-HIV	**0.92**	**0.73**	0.51	0.81	0.62
	EvoCleave	0.88	0.64	0.77	0.42	0.54
	Rognvaldsson et al.	0.9	0.7	0.69	0.62	**0.65**
	PROSPERous	0.83	0.17	0.16	1	0.27
	HIVcleave	0.56	0.29	0.29	0.45	0.35
	iProt-Sub	0.72	0.36	0.43	0.34	0.38
schillingDataset	EM-HIV	**0.96**	**0.8**	0.54	0.91	**0.68**
	EvoCleave	0.78	0.36	0.5	0.2	0.28
	Rognvaldsson et al.	0.93	0.68	0.66	0.66	0.66
	PROSPERous	0.88	0.15	0.14	0.95	0.24
	HIVcleave	0.59	0.34	0.31	0.41	0.35
	iProt-Sub	0.75	0.37	0.39	0.34	0.37

$\star$ For each dataset, the best results are bolded

**Fig. 2:**
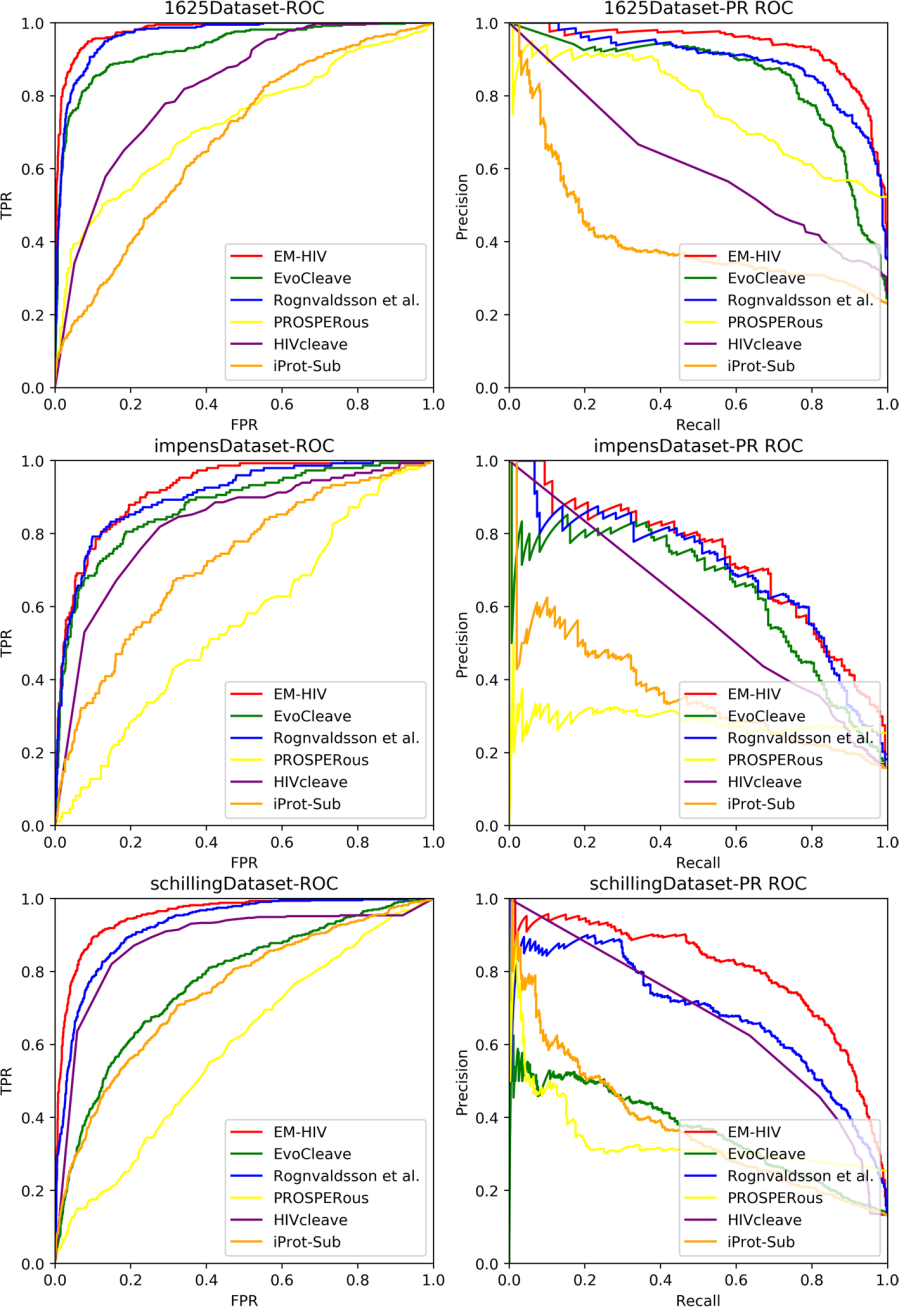
ROC and Precision–Recall curves of all prediction models

We first compare the performance of EM-HIV with other comparing methods in terms of AUC. We note that EM-HIV achieves the best performance on all three datasets, as the average AUC score obtained by EM-HIV is larger by 11%, 2%, 13%, 54% and 33% than EvoCleave, Rognvaldsson et al., PROSPERous, HIVcleave and iProt-Sub respectively. In particular, EM-HIV outperforms both EvoCleave and Rognvaldsson et al. in all cases. A possible reason for that phenomenon is that EvoCleave and Rognvaldsson et al. utilize co-evolutionary patterns and orthogonal coding respectively to generate feature vectors, while EM-HIV, on the other hand, combines these features to construct more integrated feature vectors. Our experimental results indicate that the use of features extracted from different sources can more fully exploit the sequence information of octamers, thus improving the prediction accuracy. When compared with PROSPERous and iProt-Sub that also employ a strategy of integrating multiple information sources for feature extraction, EM-HIV again demonstrates its superior performance, as it outperforms both PROSPERous and iProt-Sub in all cases. This may imply that different feature combination strategies have a different impact on the prediction performance. The ROC and Precision–Recall curves of all prediction models are presented in Fig. 2, where the ROC curves are presented on the left-hand side and the Precision–Recall curves are presented on right-hand side.

As can be seen from Table 3, the PR-AUC scores obtained by EM-HIV appear to be more frustrated when compared to its AUC scores. The fact that benchmark datasets used in our experiments are all imbalanced accounts for this phenomenon. Since the Precision–Recall analysis is more appropriate in measuring the performance of prediction models in the imbalance environment than the ROC analysis, the promising performance of EM-HIV further demonstrates its effectiveness in addressing the imbalance issue, and a conclusion could be thus made that EM-HIV has good prediction performance on imbalance datasets due to the incorporation of asymmetric bagging.

In addition, we note that the robust performance of EM-HIV in F-measure is not as obvious as AUC and PR-AUC, and it only yields the best performance on 1625Dataset and schillingDataset. To further investigate the performance of EM-HIV in terms of F-measure, a detailed analysis to the prediction results of EM-HIV is conducted. We find that many uncleavable octamers are identified with a prediction score greater than 0.5, and this fact actually results in a smaller Precision score and a higher Recall score. It could be a strong indicator that EM-HIV is much more interested in predicting cleavable octamers. By using a biased SVM with a larger value of $C_1$ as the weak learner, the resulting EM-HIV model is more biased towards the minority class, i.e., cleavable octamers.

Regarding the performance of EM-HIV in terms of runtime and peak memory, we only compare it with that of Rognvaldsson et al., as the experimental results for the other baseline prediction models are obtained from corresponding online web servers. Since Rognvaldsson et al. only adopts a linear SVM classifier with the standard orthogonal encoding scheme, it consumes less runtime and peak memory than EM-HIV. The reasons accounting for the low efficiency of EM-HIV are two-fold. First, when extracting features from substrate sequences, the determination of coevolutionary patterns requires more time for computation. Second, the asymmetric bagging strategy adopted by EM-HIV consumes more time and peak memory than only training a single linear SVM classifier.

In summary, the experimental results show that EM-HIV has a strong performance in predicting HIV-1 protease cleavage sites. It is the incorporation of asymmetric bagging and biased SVM that greatly alleviates the impact of imbalance and false-negative instances in the benchmark datasets.

### Cross data validation

To investigate the prediction performance of EM-HIV between different datasets, we additionally conduct the experiments of cross data validation, where a prediction model is trained and evaluated on independent datasets. Experimental results are shown in Table 4, and several things are worth commentary.

**Table 4:** Experimental results of cross data validation

Training set	Testing set	AUC	PR-AUC	F-measure
1625Dataset	impensDataset	0.83	0.61	0.57
	schillingDataset	0.89	0.58	0.56
impensDataset	1625Dataset	0.89	0.67	0.63
	schillingDataset	0.93	0.7	0.55
schillingDataset	1625Dataset	0.96	0.85	0.79
	impensDataset	0.91	0.76	0.68

First, we note that the performance of EM-HIV is the best by taking schillingDataset as the training data. Considering the performance of EM-HIV in 10-fold CV, EM-HIV also yields a much better performance on schillingDataset. Based on these observations, EM-HIV can produce a larger performance improvement on schillingDataset. Moreover, we also compare all octapeptides in these datasets and find that there is only a small overlap among 1625Dataset, impensDataset and schillingDataset. Hence, the promising performance of EM-HIV on schillingDataset is not caused by the shared octamers across these datasets. When compared with the other two datasets, schillingDataset is more imbalanced, i.e., a greater difference in the numbers of cleavable and uncleavable octamers. A larger imbalance degree on schillingDataset leads to a greater diversity in individual training subsets generated by the asymmetric bagging strategy. It is for this reason that the overall performance of EM-HIV is better on schillingDataset.

Second, regarding the generation of benchmark datasets, 1625Dataset is generated by mutating single amino acids in the sheared octamers, while the other two datasets are obtained from practical experiments on human proteins. As seen from the experimental results, when EM-HIV takes 1625Dataset as the training dataset, its performance on the other two datasets is the worst. Therefore, 1625Dataset may not be the most suitable training dataset for studying the substrate specificity of human proteins.

Last, we note that EM-HIV fails to perform well in terms of F-measure on all datasets. As a popular metric for binary classification problems, F-measure is the harmonic mean of Precision and Recall. In this regard, a worse F-measure performance indicates that the prediction model may have low confidence in correctly identifying cleavable octamers. To investigate the reason, we conduct an in-depth study on these datasets and find that 1625Dataset shares much less octamers with either impensDataset and schillingDataset. In particular, 1625Dataset and schillingDataset share 20 octamers, while there is no overlap between 1625Dataset and impensDataset. Hence, the features extracted from 1625Dataset have a weak predictive power on the positive instances in impensDataset and schillingDataset, thus accounting for the worse F-measure performance when we take 1625Dataset as the training set in the experiments of cross data validation.

### Impact of imbalance environment

Although biased SVM is originally proposed for positive unlabeled learning, its promising performance in imbalance environment has been verified by many studies [46–48]. When compared with traditional SVM classifier biased towards majority class, biased SVM is able to achieve good performance especially for minority class by assigning proper values to $C_1$ and $C_2$. In particular, a smaller value of $C_2$ considerably reduces the sensitivity of EM-HIV against negative samples, which belong to the majority class in our problem.

**Table 5:** Performance of EM-HIV with different classifiers in the imbalance environment

Dataset	Classifier	AUC	PR-AUC	F-measure
1625Dataset	biased SVM	0.98	0.94	0.86
	BRF	0.94	0.86	0.87
impensDataset	biased SVM	0.92	0.73	0.62
	BRF	0.91	0.67	0.47
schillingDataset	biased SVM	0.96	0.8	0.68
	BRF	0.92	0.71	0.44

**Fig. 3:**
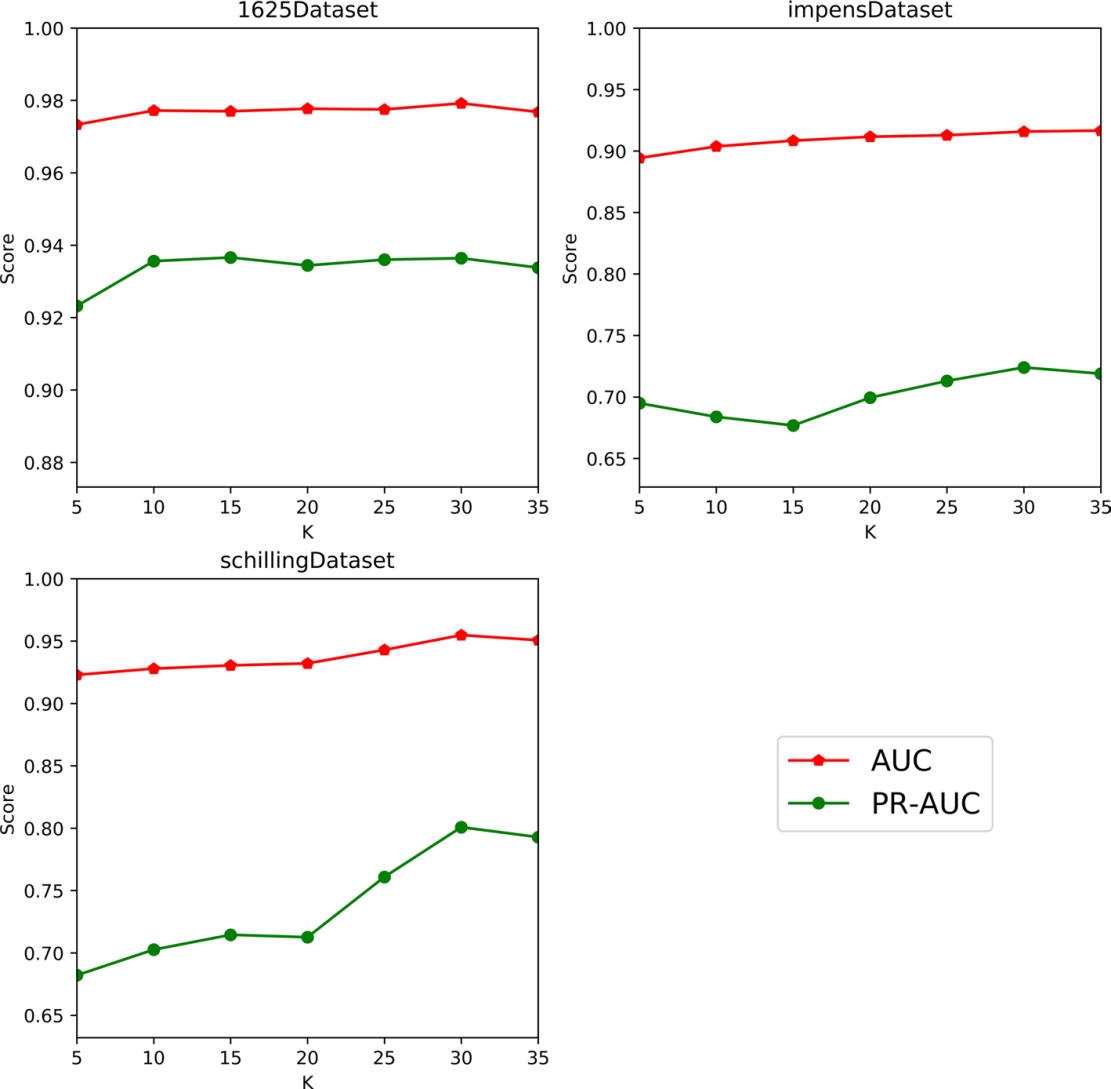
The performance of EM-HIV given different value of *K*

To verify the superiority of EM-HIV in the imbalance environment, we also evaluate the performance of EM-HIV by just replacing biased SVM with balanced random forest (BRF) [49], which is especially designed in imbalance environment. Experimental results are presented in Table 5. On average, the use of biased SVM improves the prediction performance of EM-HIV by 3.2%, 10.3% and 21.3% in terms of AUC, PR-AUC and F-measure respectively when compared with BRF. This makes biased SVM a preferred choice to address the imbalance issue in predicting HIV-1 protease cleavage sites.

### Sensitivity analysis on *K*

To investigate the impact of weak learners, we first vary the value of *K* over the set $\lbrace 5,10,15,20,25,30,35\rbrace$, and then select the value yielding the best performance of EM-HIV as the recommended number of weak learners. Regarding the sensitivity analysis on *K*, we present the experimental results in Fig. 3, and analyze them from three aspects. First, for EM-HIV, despite the relatively good robustness of AUC against the change in the number of weak learners, i.e., *K*, its PR-AUC scores appear to fluctuate with an increasing trend when *K* increases from 5 to 30. Second, the performance of EM-HIV in terms of both AUC and PR-AUC degrades when *K* is further increased to 35. This could be a strong indicator that EM-HIV may encounter the over-fitting problem for a larger number of weak learners. Last, the performance of EM-HIV is more fluctuated on impensDataset and schillingDataset than on 1625Dataset. Since both impensDataset and schillingDataset are more imbalanced than 1625Dataset, a possible reason for this phenomenon is due to the difference in the imbalance degree of these three datasets.

### Feature significance analysis

When constructing the feature vectors of octamers, we extract three kinds of features from substrate sequences, and they are amino acid identities (AAI), chemical group properties (CheP), and variable-length coevolutionary patterns (VLCoP). In order to evaluate the contributions of different features to the performance of EM-HIV, we have conducted experiments and present the average scores of AUC, PR-AUC and F-measure obtained by EM-HIV with different combinations of features in Table 6.

**Table 6:** Experimental results of feature significance analysis

Feature	Average AUC	Average PR-AUC	Average F-measure
AAI	0.94	0.77	0.69
CheP	0.91	0.69	0.63
VLCoP	0.82	0.56	0.54
AAI+CheP	0.94	0.81	0.71
AAI+VLCoP	0.94	0.79	0.7
CheP+VLCoP	0.93	0.74	0.67
AAI+CheP+VLCoP	0.95	0.82	0.72

Regarding the contributions of individual features, EM-HIV obtains its best performance when using AAI to construct the feature vectors of octamers. In this regard, the features extracted from amino acid identities are most informative for prediction, and this finding is also consistent with the results of [21]. By combining different kinds of features, we observe an improvement in the performance of EM-HIV. This could be a strong indicator that integrated feature vectors can provide more evidence to support or refute the existence of HIV-1 cleavage sites that those constructed from only one kind of features. Regarding the combination of two kinds of features, we note that the combination of amino acid identities and chemical group properties, i.e., AAI+CheP, yields the best performance. Finally, among all possible combinations of features, the consideration of all features further improves the performance of EM-HIV, but such improvement is rather limited when compared with the performance of AAI+CheP. In this regard, we believe that VLCop has the least contribution on the prediction performance of EM-HIV. The correlation between variable-length co-evolutionary patterns and the existence of cleavage sites is not as strong as amino acid identities and chemical group properties.

## Discussion and conclusion

Although a variety of machine learning models have been developed to predict HIV-1 protease cleavage sites, they are not well designed to alleviate the impacts of imbalance and false-negative octamers in the training data. Since uncleavable octamers are often artificially generated by specific strategies, the number of uncleavable octamers is far larger than that of cleavable octamers in the existing benchmark datasets, and consequently the imbalance issue severely influences the performance of prediction models. Since most machine learning models train the classifier based on the assumption of a balanced distribution of positive and negative instances, their prediction results are heavily biased towards the majority class, which is composed of uncleavable octamers in our case. In this regard, they generally obtain poor prediction performance for identifying cleavable octapeptide. On the other hand, it is possible that some unconfirmed cleavable octapeptide are falsely labeled as negative instances, and the features thus identified may confuse the classifiers to make the correct prediction.

To address these problems, we propose a novel ensemble learning model, namely EM-HIV. It first uses a comprehensive combination of three different coding schemes to construct the feature vectors of octamers. After that, it follows the idea of asymmetric bagging to resample subsets from the original training set, and trains a set of biased SVM classifiers to complete the prediction task in a more comprehensive manner. Unlike the traditional bagging idea, asymmetric bagging only resamples negative instances each time to create a more balanced training dataset. The biased SVM is selected as the weak learner to the performance of EM-HIV in predicting cleavable octamers, thereby reducing the impact of false negative data. In order to verify the effectiveness of EM-HIV, we have conducted experiments on three independent benchmark datasets. The experimental results demonstrate that the performance of EM-HIV is better than state-of-the-art prediction models.

There are two reasons contributing to the promising performance of EM-HIV for the task of HIV-1 protease cleavage site prediction. First, we extract the features of substrate sequences from different perspectives, and integrate them to construct a more expressive feature vector for each octapeptide. Second, the strategy of combining asymmetric bagging and biased SVM enhances the ability of EM-HIV against the issue of data imbalance, thus improving the performance of EM-HIV.

Regarding the future work, we would like to unfold it from three aspects. First, we are interested in employ deep learning models to extract high-quality abstract features from substrate sequences. Second, since the feature vectors of octapeptides are high-dimension, we would like to perform a feature selection process before training EM-HIV. Consequently, redundant and useless features can be disregarded by this process, thus improving the generalization ability of EM-HIV. Last, we are interested in reimplementing EM-HIV in a distributed manner for improved efficiency in terms of runtime [50].

## Data Availability

The dataset and source code can be freely downloaded from https://github.com/AllenV5/EM-HIV.
